# Rearing in a Shelved Environment Chronically Modifies Offspring Behaviour and Physiology

**DOI:** 10.1111/ejn.70199

**Published:** 2025-07-20

**Authors:** Logan J. Bigelow, Emily K. Pope, Carey G. Ousley, Ariana E. McGrattan, Debra S. MacDonald, Paul B. Bernard

**Affiliations:** ^1^ Department of Biomedical Sciences University of Prince Edward Island Charlottetown Prince Edward Island Canada

**Keywords:** behaviour, corticosterone, development, rat, rearing, stress

## Abstract

The study of environmental enrichment in rodents has primarily focused on adolescents and adults, with less information available for nursing dams and their pups. While we have previously observed some differences in behaviour and physiology of pups reared in shelved versus single‐level cages, further examination of this relationship is necessary. To understand the impact of rearing in shelved cages, we assessed various parameters in pups including body weight, ultrasonic vocalizations, hair corticosterone concentration, behaviour in the open field and elevated plus maze and spatial working memory in the spontaneous alternation task. In addition, dams were assessed in the open field and elevated plus maze to identify any changes in stress‐related behaviour. As adults, rats reared in enriched cages had significantly lower body weights, higher hair corticosterone concentrations and spent less time in the open arms of the elevated plus maze compared to those in standard cages. Additionally, rats reared in enriched cages emitted a lower number of frequency‐modulated calls. In agreement with the behaviour observed in their pups, dams housed in enriched cages spent significantly less time in the centre of the open field. The results indicate that there are long‐term changes in behaviour and physiology based upon different rearing conditions, reinforcing the importance of considering rearing environment when planning studies of a developmental nature. Furthermore, determining optimal rearing conditions will not only improve laboratory animal welfare but also improve reproducibility in animal research through the standardization of rearing conditions across institutions and laboratories.

## Graphical abstract

Rats reared in an enriched cage featuring a shelf and increased vertical height exhibited significant differences in physiology and behaviour, including lower body weight, higher hair corticosterone, lower production of 50 kHz ultrasonic vocalizations and increased anxiety‐related behaviour, compared to rats reared in standard cages. Further research examining the consequences of varied rearing environment will improve both animal welfare and experimental reproducibility in rodent research. Image created in Biorender (2025).

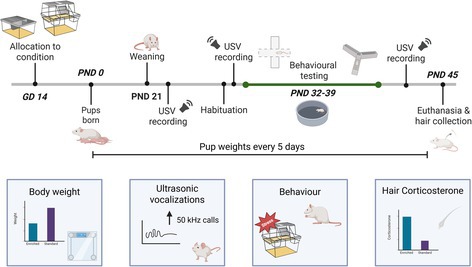

## 1 Introduction

Over the past decade, there has been an increasing interest in the impact of environmental enrichment on laboratory animals, especially considering modifications in the guidelines for the care and use of laboratory animals (National Research Council et al. 2010; Ratuski and Weary 2022; Percie du Sert et al. 2020). While many enrichment studies are focused on modifications to the housing environment of adolescent or mature rodents (Simpson and Kelly 2011), the enrichment of nursing rodents has garnered less attention. Although some studies have investigated the implications of different forms of environmental enrichment during rearing (Baldini et al. 2013; Bigelow, Pope, et al. 2022; Hutchinson et al. 2012; Korgan et al. 2016; Ratuski and Weary 2021), most of these studies focus on maternal impacts rather than long‐term consequences in the offspring. A more comprehensive understanding of the effects of enrichment is not only beneficial in improving animal welfare but also essential in understanding the factors that contribute to experimental reproducibility (Bayne and Würbel 2014; Loss et al. 2021).

The nursing environment of laboratory rats is unique compared to that of rats in nature, particularly as it relates to the confinement of the mother and pup accessibility to the dam. Within nature, dams have the opportunity to leave the nest and escape the demands of their pups at their own discretion, whereas, within a laboratory, the dam is confined to the same area as her pups at all times (Ladd et al. 2000). It has been demonstrated that, when permitted, dams tend to spend less time with their pups later in development (Grota and Ader 1969), with various studies citing the welfare benefits of allowing mothers a temporary reprieve from their pups (Dawson et al. 2013; Ratuski and Weary 2021; Weaver et al. 2016). A recent study reported that dams nursing in a shelved environment increasingly utilized the shelf as the pups became older, subsequently showing a greater positive affect and a decrease in total nursing time (Ratuski and Weary 2021). Such changes in dam behaviour are likely to result in modifications to the behaviour and physiology of the offspring. Maternal separation studies have previously shown behavioural and physiological changes in both mother and offspring following varying durations of separation (Aisa et al. 2008; Bölükbas et al. 2020; Lundberg et al. 2017; Ojha et al. 2024; Roque et al. 2014). Such studies also cite that not only does the duration of separation influence the animals but also whether the separation occurred early versus later in development (Ojha et al. 2024). Our previous findings suggested that rats reared in standard cages had reduced anxiety‐like behaviour compared to those reared in shelved cages (Bigelow, Pope, et al. 2022); however, the preliminary nature of these results did not allow for the control of litter effects. Despite evidence that litter to litter variation influences experimental outcomes, it is rarely reported; a recent review shows that litter effects were only accounted for in approximately 12% of papers focused on rodent neurodevelopmental disorders (Jiménez and Zylka 2021). To improve the reliability of our previous findings, litter was considered within the current study.

The effects of environmental enrichment on rodents may be assessed through both physiological and behavioural parameters. Corticosterone, one of the primary stress hormones in rats (Scorrano et al. 2015), is incorporated into various biological samples following a stressor and serves as a frequently utilized metric of animal stress (J. S. Meyer and Novak 2012). While blood corticosterone is often assessed (Vahl et al. 2005), blood collection is stressful to the animal (N. Meyer et al. 2020; Tuli et al. 1995), indicative of acute stress (Vahl et al. 2005) and confounding if repeatedly performed (Abelson et al. 2005). Faecal matter instead provides a snapshot of stress over a longer time period (Bamberg et al. 2001), but the circumstances of collection can be unreliable and potentially confounding. Such shortcomings may be well addressed by analysis of hair corticosterone (Scorrano et al. 2015), an effective and non‐invasive collection method (Uarquin et al. 2016).

Behavioural tests are often utilized in conjunction with physiological assessments to determine the affective state of laboratory animals. For instance, the open field and elevated plus maze are two of the most common tests to assess anxiety‐like behaviour in rodents (de Figueiredo Cerqueira et al. 2023). Unfortunately, recent studies have frequently cited contradictory results using various behavioural tests of anxiety (Snyder et al. 2021); thus, additional analyses can assist in gaining a broader perspective of the overall well‐being of the animal. Analysis of ultrasonic vocalizations (USVs) serves as an additional non‐invasive assessment of affective state in rats. USVs are sounds produced by rats to communicate; generally, 50 kHz calls relay positive emotion while 22 kHz calls relay negative emotion. Accordingly, various classification schematics have been developed ranging in complexity from a generalized model consisting of three sub‐types of 50 kHz calls (S. M. Brudzynski 2021) to a complex 14 call schematic (Wright et al. 2010). Assessment of USVs using elaborate schematics can be cumbersome and subjective (Burke et al. 2017); such shortcomings can thus be addressed through using simpler schematics without any definitive loss of information. Additionally, cognitive capacity, particularly spatial working memory, may be assessed with the spontaneous alternation task (Frick et al. 2003; Galeano et al. 2015; Simpson and Kelly 2011).

The aim of this study was to explore the consequences of rearing in a shelved environment through assessment of changes in body weight, behaviour in an array of tasks, production of ultrasonic vocalizations and hair corticosterone concentration. Furthermore, behavioural tests in the dams near time of offspring weaning sought to determine any modifications in dam behaviour that may be responsible for long‐term effects observed in their offspring.

## 2 Methods

### 2.1 Animal Husbandry

**FIGURE 1:**
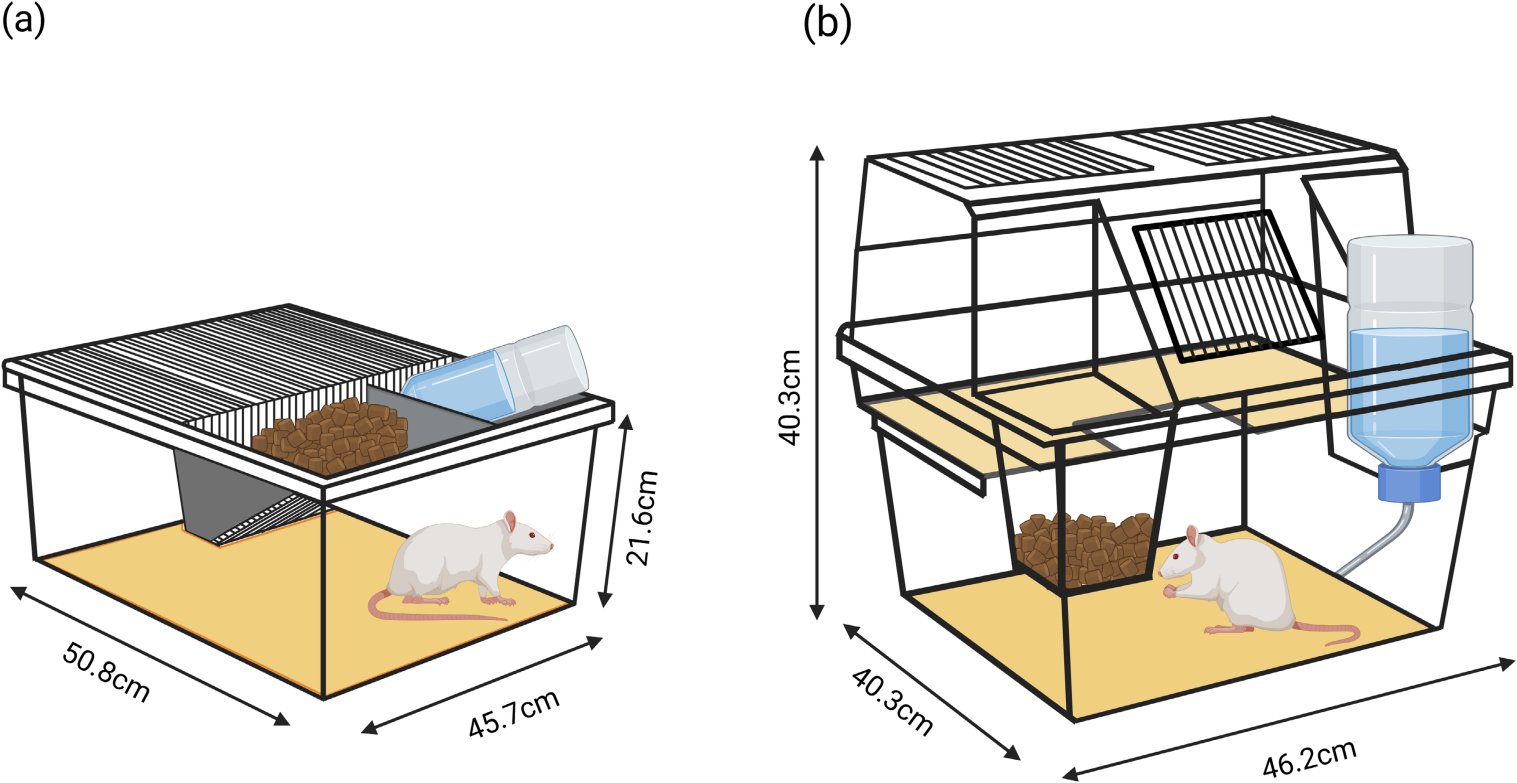
Housing conditions. Dams were either housed in (a) standard single‐level cages or (b) enriched cages featuring a shelved area that allowed the dam to escape her pups. Image created in Biorender (2025).

Sprague–Dawley (CD) dams, at approximately gestational day (GD) 14, were obtained from Charles River Laboratories (Saint‐Constant, QC, Canada) in two cohorts of 10. Upon arrival, each cohort of dams was randomly allocated to standard single‐level cages with dimensions of 50.8 × 40.6 × 21.6 cm (Ancare Corporation, Bellmore, NY, USA) or GR1800 Double Decker cages, termed enriched cages throughout the study, with dimensions of 38.1 × 30.5 × 39.4 cm (Tecniplast, Montreal, QC, Canada) (Figure 1). Following arrival, dams were left undisturbed aside from regular cage checks until pups were born on gestational day 21, which was subsequently termed post‐natal day (PND) 0. Pups were pseudo‐randomly culled to a maximum of 10 per litter with consideration of sex in a 1:1 ratio. The first cohort was composed of 99 pups (one litter of nine pups, and nine litters of 10 pups) and the second cohort was composed of 95 pups (two litters of eight pups, one litter of nine pups, and seven litters of 10 pups). Twenty litters were used to ensure an adequate sample size with consideration of litter effects (Lazic and Essioux 2013). On PND 21, animals were weaned and reallocated to standard cages in groups of two with consideration of sex. Cages were changed twice per week and bedded with Hardwood Beta Chips (North‐Eastern Products, Warrensburg, NY, USA). Room conditions were maintained between 19°C–22°C, 30%–52% humidity and 70–80 dB ambient sound with a reverse light–dark cycle (lights on between 18:00 and 06:00 h). All animals were maintained on an ad libitum diet of Laboratory Rodent Diet 5001 (LabDiet, Saint Louis, MO, USA) and water. All testing occurred during the dark phase of the light–dark cycle where red lights were utilized as necessary given the dark phase is when rodents are more active (Roedel et al. 2006). All procedures performed during this study were conducted in accordance with the guidelines of the Canadian Council on Animal Care and approved by the University of Prince Edward Island Animal Care Committee protocol #21‐032.

### 2.2 Assessment of Offspring and Dams

All pups were weighed every 5 days throughout the duration of the study (PND 5–40) to determine any differences in weight. Ultrasonic vocalization recordings were performed throughout development on PND 22, 31, and 40. Each recording was obtained from groups of two rats of the same sex within their home cage; thus, the cage served as the statistical unit. Prior to testing, rats were transferred to the behavioural suite within their home cage and left undisturbed for 5 min. Following the 5‐min habituation period, a recording was obtained using a CM 16 ultrasonic microphone (Avisoft Bioacoustics, Glienicke/Nordbahn, Germany) placed approximately 5 in. above the cage. Vocalizations were recorded using Avisoft UltraSound Gate (Avisoft Bioacoustics, Glienicke/Nordbahn, Germany) for a duration of 5 min and analysed manually using the open source software Deep Squeak (Coffey et al. 2019). The number of calls produced within each 5‐min trial was determined, and all calls were categorized according to frequency as either 50‐ or 22‐kHz. The 50‐kHz calls were further categorized into three groups: trill‐type calls, flat‐type calls and frequency‐modulated calls (S. Brudzynski 2015). Additional criteria for call classification included a minimum duration of 4 ms per call, where components of a call had to be less than 10 ms apart; if components were greater than 10 ms apart, it was classified instead as two distinct calls. Flat calls were required to have a slope between −200 and 200 kHz/s, and trills were required to have at least two bidirectional changes and were usually greater than 9 ms in duration.

All behavioural testing occurred between PND 32 and 39, with habituation to the testing procedures taking place the week prior to testing (PND 27–31). Testing within the elevated plus maze took place on PND 32–33, testing within the open field took place on PND 34–37, and testing in the spontaneous alternation task took place on PND 38–39. All animals from each litter were tested in all the behavioral tests to examine intralitter variation. Animals were habituated by transporting each cage to the testing suite for a period of 5 min and then returning the cage to the colony room on three separate days. The open field apparatus consisted of a circular arena (inner diameter: 91.5 cm, side height: 50.8 cm). The elevated plus maze consisted of two enclosed arms (side height: 30.5 cm) and two open arms, each positioned opposite one another (arm length: 111.8 cm, arm width: 10.2 cm), oriented in a plus configuration elevated off the floor (height: 83.8 cm). The spontaneous alternation apparatus consisted of three arms, distributed at angles of 120° and connected by a centre area (arm length: 57.0 cm, arm width: 15.0 cm, side height: 30.0 cm). At the start of each trial, an animal was randomly selected and transported to the testing suite in a transfer cage. For the open field, the animal was placed in the centre of the arena and allowed to freely explore for a period of 10 min, during which a video recording was obtained. For the elevated plus maze, the animal was placed in the centre of the maze facing an open arm, and for the spontaneous alternation task, the animal was placed in an arm facing away from the centre; in both tasks, the animal was allowed to freely explore for a 5‐min period during which a video recording was obtained. At the end of each test, the rat was removed from the maze, and the maze was cleaned with Virkon disinfectant (Quebec, Canada) before the next animal was assessed. All video recordings were obtained using a HiSeeU infrared camera at a wavelength of 590 nm (London, United Kingdom). The primary measures of interest within the open field and elevated plus maze were time spent in the centre and time spent in the open arms, respectively. The primary measure of interest in the spontaneous alternation task was percentage of alternations determined by: [(number of alternations) / (triads)] × 100, where the number of alternations is when all three arms were visited before revisitation of an arm, and the number of triads represents the total number of arm entries minus two. Distance travelled within all behavioural tests was also assessed to account for any variation in locomotion. Video recordings were analysed using Noldus Ethovision XT (Wageningen, Netherlands). Some videos were excluded from analysis due to corrupt video files.

Prior to weaning on PND 21, dams were assessed in the elevated plus maze (PND 20) and open field (PND 21) using the same procedures as previously described for the offspring behavioural testing. In short, each dam was randomly selected and placed in a transfer cage to be transported to the testing suite. For the elevated plus maze, each dam was placed in the centre of the maze facing an open arm and allowed to explore freely for 5 min, and for the open field, each dam was placed in the centre of the maze and allowed to explore freely for a period of 10 min while a video recording was obtained. Dams were subsequently returned to their pups in the home cage following each test.

On PND 45, rats were anaesthetised using isoflurane beginning at 1% and increasing at 1% intervals every 30 s until the animal was unconscious for at least 30 s. The oxygen flow rate was 1.5 L/min. The dorsal surface of each rat was subsequently shaved. A large sample of hair was taken to account for any local variation in hair growth and/or corticosterone incorporation. Hair samples were stored at −80°C for future analysis. A male and female hair sample from each litter was randomly selected and retrieved from the −80°C freezer; 200 mg of hair was added to 15 mL of isopropanol and gently mixed on a rocker for 3 min. The sample was then centrifuged at 1500 ×*g* for 1 min, and the supernatant was removed. This step was repeated for a total of 3 washes. The hair was then dried at 37°C for ~30 min, or until completely dry. The hair was then placed in a stainless‐steel mortar, and liquid nitrogen was poured over the sample. The sample was then crushed with a pestle while the liquid nitrogen evaporated. This process was repeated (2–3×) until the hair was crushed into a powder. Next, 100 mg of powdered hair was added to 5 mL of methanol, and the tube was placed on the rocker and left overnight. The following day, the tubes were removed from the rocker and centrifuged for 10 min at 6000 ×*g*, and 3 mL of supernatant was removed and placed into a 15 mL tube. The tubes were attached to a nitrogen evaporator and dried under a slow stream of nitrogen gas until all the liquid was gone (1 h). The extract was reconstituted in 200 μL of ELISA assay buffer and stored at −20°C. Sample supernatants were diluted 1:4, and corticosterone concentration was determined with commercial ELISA kits (Arbor Assays, DetectX Corticosterone Multiformat ELISA kit, K014). The upper and lower limits of detection for corticosterone were 39.1 pg/mL and 10,000 pg/mL, respectively, with an expected sensitivity of 20.9 pg/mL for 50 μL and 14.4 pg/mL for 100 μL samples.

### 2.3 Statistical Analysis

All data were analysed using Jamovi version 2.3 for Windows (Jamovi Software), and graphs were generated using GraphPad Prism 9 version 6.01 for Windows (GraphPad Software). Assessment of dams in the behavioural tests was performed via unpaired *t*‐tests. For the analysis of offspring weights, behavioural tests and home‐cage ultrasonic vocalizations, a linear mixed model was utilized with litter included as a random effect to account for similarities between pups from the same litter (Jiménez and Zylka 2021). For assessment of weight as well as for all the behavioural tests, treatment and sex were used as predictors with dependent variables of weight, time in centre (for the open field), time in open arms (for the elevated plus maze), percent alternations (for the spontaneous alternation task) and distance travelled (all behavioural tests). For the assessment of home cage ultrasonic vocalizations, treatment, sex and time (day 22, 31 or 40) were used as predictors with the number of each call type as the dependent variable. Assessment of hair corticosterone was determined via a two‐way ANOVA using treatment and sex as factors. Post hoc analysis consisted of unpaired *t*‐tests, and significance in all comparisons was determined by *p* ≤ 0.05.

## 3 Results

### 3.1 Offspring Weights

**FIGURE 2:**
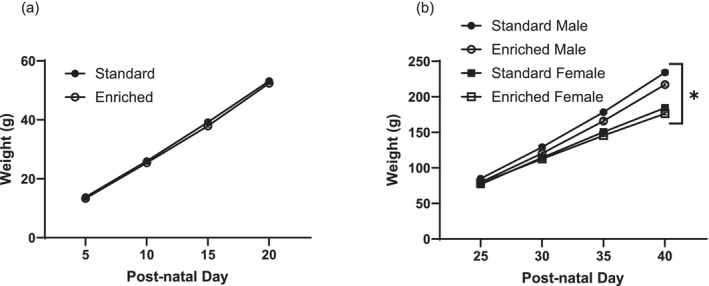
Weights. Weight of all pups throughout the duration of the study pre‐weaning (a) and post‐weaning (b). Pre‐weaning, pups were housed with the dam in either standard or enriched cages, while post‐weaning, pups were all reallocated to standard cages. Data given as mean ± SEM, *n* = 44–52, * indicates significance of *p* < 0.05. Pups reared in enriched cages had significantly lower weights (*p* = 0.026).

When assessing the weight of pups throughout the study, there was no sex effect (*F*_1,18_ = 0.0029, *p* = 0.96), but there was an effect for time (*F*_3,25_ = 1200, *p* < 0.001) and treatment (*F*_1,25_ = 5.8, *p* = 0.024) (Figure 2). A likelihood ratio test for the random effect of litter indicated a significance for time (*p* < 0.001) and sex (*p* < 0.001) but no significance for treatment (*p* = 0.35). Rats reared in enriched cages weighed significantly less than those in standard cages (*t*_20_ = 2.4, *p* = 0.026). Post hoc analysis indicated significant treatment differences in weight on both PND 35 (*p* < 0.001) and PND 40 (*p* < 0.001), indicative of long‐lasting changes.

### 3.2 Offspring Home‐Cage Ultrasonic Vocalizations

**FIGURE 3:**
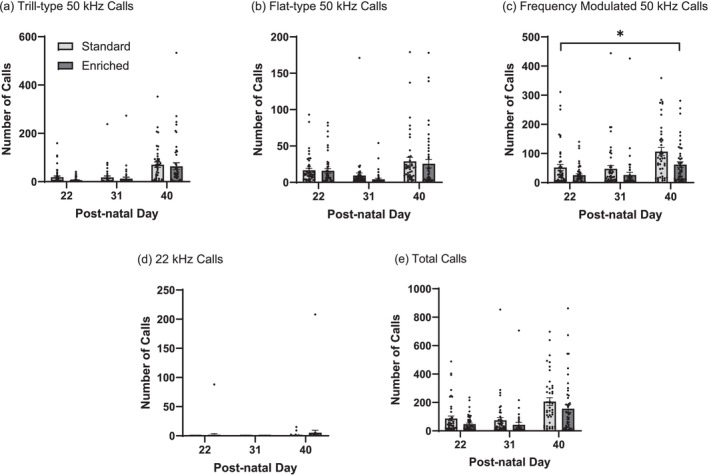
Home‐cage ultrasonic vocalizations. The number of 50‐kHz calls were analysed on post‐natal days (PND) 22, 31 and 40, and classified into three categories including (a) trill‐type, (b) flat‐type and (c) frequency modulated calls. The total number of 22‐kHz calls (d) as well as the overall total number of calls (e) produced were also determined for both rats reared in standard versus enriched cages. Data shown as mean ± SEM*, n* = 47–49, *Indicates significance *p* < 0.05. Rats reared within enriched cages produced a lesser number of frequency‐modulated calls compared to those in standard cages (*p* = 0.049).

Assessment of the number of trill‐type 50 kHz calls indicated an effect for day (*F*_2,36_ = 11, *p* < 0.001), but not for sex (*F*_1,22_ = 1.5, *p* = 0.23) or treatment (*F*_1,21_ = 0.80, *p* = 0.38) (Figure 3a). A likelihood ratio test for the random effect of litter indicated no significance (treatment: *p* = 0.94, sex: *p* = 0.53). Assessment of the number of flat‐type 50 kHz calls indicated an effect for day (*F*_2,27_ = 8.3, *p* = 0.002), but not for sex (*F*_1,37_ = 0.27, *p* = 0.61) or treatment (*F*_1,18_ = 0.30, *p* = 0.59) (Figure 3b). A likelihood ratio test for the random effect of litter indicated no significance (treatment: *p* = 0.90, sex: *p* = 0.96). Assessment of the number of frequency‐modulated 50 kHz calls indicated an effect for day (*F*_2,22_ = 9.6, *p* < 0.001) and treatment (*F*_1,17_ = 4.5, *p* = 0.049), but not for sex (*F*_1,30_ = 1.4, *p* = 0.25) (Figure 3c). A likelihood ratio test for the random effect of litter indicated no significance (treatment: *p* = 0.59, sex: *p* = 0.96). Assessment of the number of 22 kHz calls indicated no effect for day (*F*_2,36_ = 0.71, *p* = 0.50), sex (*F*_1,19_ = 0.93, *p* = 0.35) or treatment (*F*_1,29_ = 1.6, *p* = 0.21) (Figure 3d). A likelihood ratio test for the random effect of litter indicated no significance (treatment: *p* = 0.91 sex: *p* = 0.055). Assessment of the total number of calls produced indicated an effect for day (*F*_2,36_ = 15, *p* < 0.001), but no effect for sex (*F*_1,39_ = 1.3, *p* = 0.26) or treatment (*F*_1,21_ = 2.5, *p* = 0.13) (Figure 3e). A likelihood ratio test for the random effect of litter indicated no significance (treatment: *p* = 0.84, sex: *p* = 0.92). Post hoc analysis determined that rats reared within enriched cages produced a lesser number of frequency‐modulated calls compared to those in standard cages (*t*_17_ = 2.1, *p* = 0.049) (Figure 3c).

### 3.3 Offspring Behavioural Testing

**FIGURE 4:**
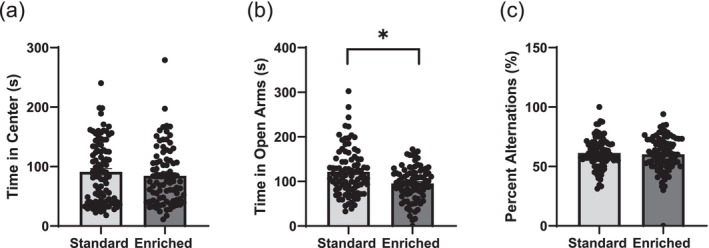
Offspring behavioural testing. Time spent in the centre of the open field (a), time spent in the open arms of the elevated plus maze (b) and percent alternations in the spontaneous alternation task (c) for rats reared in standard cages compared to rats reared in enriched cages. Data given as mean ± SEM, *n* = 90–97, *Indicates significance of *p* < 0.05. Rats reared in enriched cages spent significantly less time in the open arms of the elevated plus maze (*p* = 0.028).

When assessing time in the centre of the open field, there was no treatment effect (*F*_1,17_ = 0.23, *p* = 0.64) and no sex effect (*F*_1,45_ = 0.04, *p* = 0.85) (Figure 4a). A likelihood ratio test for the random effect of litter indicated no significance for treatment (*p* = 0.78) or sex (*p* = 0.90). When assessing time in the open arms of the elevated plus maze, there was a treatment effect (*F*_1,15_ = 5.9, *p* = 0.028), but no sex effect (*F*_1,20_ = 0.36, *p* = 0.56) (Figure 4b). A likelihood ratio test for the random effect of litter indicated no significance for treatment (*p* = 0.57) or sex (*p* = 0.67). When assessing percent alternations in the spontaneous alternation task, there was no treatment effect (F_1,23_ = 0.19, *p* = 0.67) and no sex effect (F_1,48_ = 0.14, *p* = 0.71) (Figure 4c). A likelihood ratio test for the random effect of litter indicated no significance for treatment (*p* = 0.86) or sex (*p* = 0.98). Distance travelled in the open field test yielded no treatment effect (*F*_1,18_ = 0.08, *p* = 0.78) and no sex effect (*F*_1,18_ = 1.1, *p* = 0.32). Distance travelled in the elevated plus maze yielded no treatment effect (*F*_1,18_ = 0.11, *p* = 0.75) and no sex effect (*F*_1,16_ = 0.0019, *p* = 0.97). Distance travelled in the spontaneous alternation task yielded no treatment effect (*F*_1,14_ = 0.35, *p* = 0.56) and no sex effect (*F*_1,21_ = 0.12, *p* = 0.74). No litter effects were observed for distance travelled within any behavioural tests.

### 3.4 Offspring Hair Corticosterone

**FIGURE 5:**
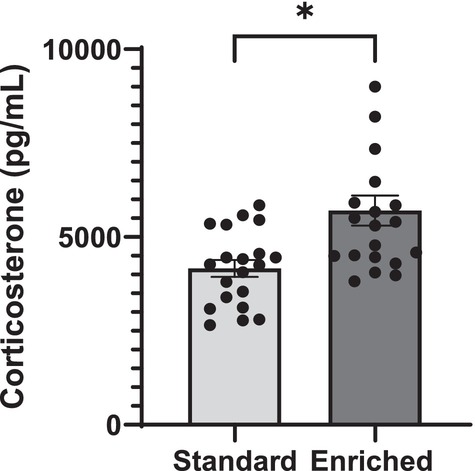
Hair corticosterone. Hair corticosterone concentration measured via commercial ELISA kits in rats reared in standard versus enriched cages. Data given as mean ± SEM, *n* = 20, *Indicates significance of *p* < 0.05. Rats reared in enriched cages had significantly greater concentrations of corticosterone compared to those reared in standard cages (*p* = 0.002).

When assessing hair corticosterone, there was a treatment effect (*F*_1,36_ = 11, *p* = 0.002), but no sex effect (*F*_1,36_ = 0.68, *p* = 0.41) (Figure 5). Post hoc analysis revealed that rats reared in enriched cages had significantly greater concentrations of corticosterone compared to those reared in standard cages (*t*_38_ = 3.4, *p* = 0.002).

### 3.5 Dam Behavioural Testing

**FIGURE 6:**
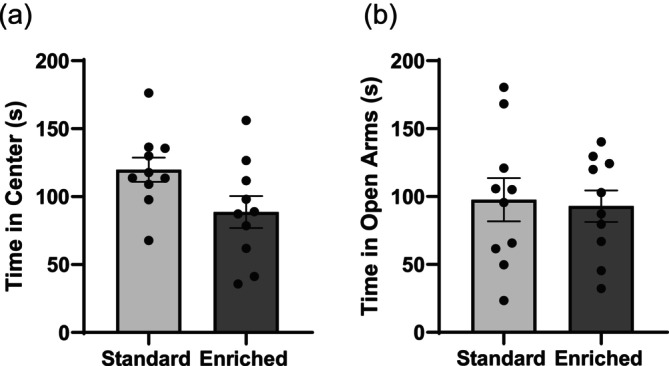
Dam behavioural testing. Time spent in the centre of the open field (a), and time spent in the open arms of the elevated plus maze (b) for dams housed in standard cages compared to those housed in enriched cages. Data given as mean ± SEM, *n* = 10, *Indicates significance of *p* < 0.05. Dams housed in standard cages spent more time in the centre of the open field compared to those housed in enriched cages (*p* = 0.049).

Dams housed in standard cages spent more time in the centre of the open field compared to those housed in enriched cages (*t*_18_ = 2.1, *p* = 0.049) (Figure 6a). Time spent in the open arms of the elevated plus maze yielded no treatment effects (*t*_18_ = 0.24, *p* = 0.81) (Figure 6b). No significant differences in distances travelled for the open field (*t*_18_ = 0.03, *p* = 0.98), or for the elevated plus maze (*t*_18_ = 0.02, *p* = 0.98), were observed.

## 4 Discussion

During the assessment of the physiological and behavioural impacts of rearing in an enriched versus standard cage, it was determined that rats reared in enriched cages had significantly lower body weight, elevated hair corticosterone concentrations, spent less time in the open arms of the elevated plus maze, and emitted fewer frequency modulated calls. Additionally, dams housed in enriched cages spent significantly less time in the centre of the open field.

We previously showed a trend toward lower body weight in pups reared in enriched cages compared to those reared in standard cages, with male rats specifically showing a significant difference in body weight (Bigelow, Pope, et al. 2022). Analysis of a larger sample size with consideration of litter effects within the current study indicated a significant difference in body weight for both male and female animals (Figure 2). The likelihood ratio test for the effect of litter on body weight did not yield any significant differences for treatment but was significant for both sex and time, indicating that litter‐to‐litter variation plays a significant role in pup body weight. Our previous study (Bigelow, Pope, et al. 2022) only reported significant differences in body weight for males, which may be due to increased inter‐litter variation in females masking the potential treatment effect, highlighting the importance of controlling for and assessing litter variation.

Various studies have previously cited the impact of litter size on offspring weight (Crozier and Enzmann 1935; Young 2002) with some studies citing that differences in maternal care are likely the cause of long‐term behavioural and physiological changes in offspring (Enes‐Marques and Giusti‐Paiva 2018). Similarly, the treatment differences observed within the present study are expected to be due to differences in maternal care. A similar study assessing dams housed in cages with loft access reported a decrease in both passive and total nursing time (Ratuski and Weary 2021); thus, it is speculated that decreased nursing behaviour may result in pups with a lower body weight. Other studies have shown changes in maternal behaviour as a result of different forms of enriched housing. For instance, dams housed in cages containing an area that allows burrowing demonstrated lower passive nursing but increased active nursing (Korgan et al. 2016), providing further evidence that changes in nursing behaviour may be due to differences in housing arrangements. Future studies should further examine the relationship between nursing behaviour and body weight throughout development within cages containing a lofted or shelved area.

Interestingly, the difference in body weight persisted post‐weaning, even becoming more pronounced over time (Figure 2b). It is relevant to note that the placement of the food hopper may have also contributed to the observed difference in body weight; in enriched cages, the food hopper was positioned within the cage near ground level whereas in standard cages, food was positioned in a rack at the top of the cage (Figure 1). At weaning, all animals were reallocated to standard cages; therefore, those previously housed in enriched cages had to acclimate to the new positioning of their food. It is possible that rats reared in enriched cages exhibited differences in food consumption due to differences in accessibility of the food hopper or that the altered placement of the food hopper may have changed the pups' demand on the mother or the mother's care for her offspring, consequently altering nursing behaviour. Regardless of the potential cause, observed differences in body weight persisted throughout the duration of the study, providing evidence of the long‐term repercussions of varied rearing environment.

The use of home‐cage USV assessment is a relatively new concept. While we previously assessed USVs in individual rats reared in either standard or enriched cages during traditional behavioural assays (Bigelow, Pope, et al. 2022), the USVs produced by rats in isolation appear to lack the richness observed in social scenarios. In contrast, recording USVs within the home‐cage captures the social interactions of the animals, allowing for ongoing assessment of rat behaviour with minimal disturbance. We previously observed differences in home‐cage USVs of adult rats under different housing scenarios (Bigelow, Cohen, et al. 2022); however, a benefit of assessing home‐cage USVs following enrichment is the ability to control microphone placement as well as other cage variables. Within this study, pups reared in standard cages produced a significantly greater number of frequency‐modulated calls compared to those reared in enriched cages; no other differences in USV production were observed (Figure 3). Frequency‐modulated calls are more commonly attributed to rewarding events or situations of highly positive arousal, whereas flat‐type calls are attributed to instances of social contact and coordination (Burgdorf et al. 2011; Seidisarouei et al. 2021; Wöhr and Schwarting 2008). The lower number of frequency‐modulated calls emitted by animals reared in enriched cages within this study may therefore be interpreted as a decrease in positive affective state; however, given the relatively novel nature of USV assessment and limited information available regarding home‐cage USVs as a consequence of enrichment conditions, additional studies examining this relationship are necessary. Previous studies have shown that rodents respond differently to the playback of USVs when housed under different enrichment scenarios. Physical enrichment was determined to reduce approach behaviour in response to the playback of 50 kHz calls while social enrichment was found to increase approach behaviour (Brenes et al. 2016). Consequently, it is likely that the assessment of home‐cage USVs cannot be simply interpreted as representing positive versus negative affective state but rather must be utilized to better understand rodent interactions with one another under different conditions. Various studies continue to characterize different rodent vocalizations and assess how to best utilize USVs as a metric of animal welfare (Lupfer et al. 2023); thus, understanding differences in rodent USVs as a result of changing environment is crucial to furthering the use of USVs as a novel assessment of rodent welfare. In addition, assessing the volume of calling throughout the lifespan of the rats, including the early period, where 40‐kHz pup isolation induced USVs may be assessed, could provide further insight into the nature of calls produced later in adulthood (Schwarting and Wöhr 2018).

It was previously observed that rats housed in standard cages spent significantly more time in the centre of the open field compared to those housed in enriched cages (Bigelow, Pope, et al. 2022); however, within the current study, no differences were observed (Figure 4a). The previous study utilized a smaller number of litters and did not control for litter effects during analysis, potentially accounting for the observed differences. Although no differences in behaviour were observed in the open field, rats reared in enriched cages spent significantly less time in the open arms of the elevated plus maze (Figure 4b), indicative of increased anxiety. While various tests of anxiety‐like behaviour, such as the open field and elevated plus maze, have previously been shown to disagree (de Figueiredo Cerqueira et al. 2023), a recent meta‐analysis assessing the reliability of 17 behavioural tests of anxiety using an array of anxiolytic compounds has shown the elevated plus maze and light–dark box to be the most reliable tools for the assessment of anxiety (Rosso et al. 2022). While further refinement of these tests and a thorough assessment of the reliability of these tests in rats as opposed to mice is warranted, it was expected that the elevated plus maze portrayed a more reliable metric of anxiety as compared to the open field within this study. Such findings appear to contradict previous studies, which found that rodents reared in enriched conditions exhibited decreased anxiety‐like behaviour in the elevated plus maze (Baldini et al. 2013). The term ‘enriched environment’ is highly variable among different studies. The enriched environment within the current study consisted of a shelf in addition to increased vertical height; however, alternate enrichment strategies may include multiple shelves, running wheels, various forms of shelter, novel objects or some combination thereof (Ciucci et al. 2007). The differences observed between studies are likely due to different forms of enrichment and their varied impact on rodent behaviour and physiology. It is reasonable to justify that drastic changes in environment would result in more profound differences compared to minor modifications in environment.

Considering the pups have greater mobility toward the weaning date and are able to begin utilizing the complexities of an enriched environment, it was hypothesized that this may impart a stronger spatial working memory. Previous studies have reported that rodents housed within an enriched environment demonstrate improvements in learning and memory (Simpson and Kelly 2011). Environmental enrichment introduced later in life has even been found to decrease age‐related spatial memory declines in both males and females (Frick et al. 2003). Despite previous studies finding a significant relationship between environmental enrichment and memory, no treatment differences were observed in the spontaneous alternation task within this study (Figure 4c). Given the pups were only able to begin exploring the upper shelf a few days prior to weaning, it was expected that this limited experience had a negligible impact on the pups. It should be noted that the continuous spontaneous alternation task was chosen as opposed to the discrete since rats maintain high arm entry rates when allowed to continuously explore; however, the inter‐trial interval between arm entries is not controlled (Hughes 2004) potentially complicating interpretation. Alternatively, previous work has cited the impairment of learning and memory as a consequence of maternal stress (Fu et al. 2022). Within the current study, dams were obtained on GD 14 and allocated to either standard or enriched cages; it is therefore possible that the maternal stress associated with transfer to a novel environment may have overshadowed any potential improvements in learning and memory within the offspring reared within the enriched condition. Additionally, it should be noted that while sex differences in behaviour and performance in certain tasks has been previously documented based upon rearing environment (Seymoure et al. 1996), no sex differences were observed for any behavioural task within the current study. There was also no significant difference observed in distance travelled throughout any of the behavioural tests indicating that differences in rearing environments did not result in changes in activity or locomotion of the offspring.

Recently, researchers have been highlighting the importance of controlling for litter effects to improve the reproducibility of animal research, citing that litter can potentially account for up to 60% of variability in commonly studied rodent phenotypes (Jiménez and Zylka 2021). Within the current study, the random effect of litter was not statistically significant for treatment or sex when assessing both home‐cage ultrasonic vocalizations as well as the various behavioural outcomes, meaning that littermates were not significantly more similar to one another than to animals from a different litter (in terms of the parameters assessed). Previous protocols employed for developmental studies used litter as the statistical unit by either taking one male and one female from each litter or averaging data from all littermates (Golub and Sobin 2020; Vorhees and Williams 2020). Such methods rely on the assumption that littermates are significantly similar to one another and thus justify that one animal may be representative of the litter as a unit (Golub and Sobin 2020). Within this study, littermates were not found to be significantly similar, and thus such an assumption should not be implied. Utilization of the previous model where only one animal per litter is assessed would therefore not accurately represent the true outcome in this case and neglect the use of valuable data that could have been included within the study design (Golub and Sobin 2020).

Prior investigations revealed no differences in faecal corticosterone concentrations between animals reared in standard versus enriched cages (Bigelow, Pope, et al. 2022); however, rats reared in enriched cages had increased hair corticosterone concentrations within the current study (Figure 5). While elevated corticosterone concentrations within rodents are frequently associated with a negative connotation within the literature, assessment of corticosterone is more broadly representative of the activation of the hypothalamic–pituitary–adrenal axis and may not necessarily be indicative of negative stress (Jimeno et al. 2018). In essence, the elevated corticosterone concentrations observed in the offspring reared in enriched cages may be interpreted as a response of their adaptation to a change in environment rather than indicative of increased stress in these animals. While hair corticosterone analysis represents a relatively novel method of assessment with fewer studies utilizing this metric compared to more traditional methods such as faecal and blood analysis, hair glucocorticoid analysis has recently been gaining popularity due to its non‐invasive and reliable nature (Scorrano et al. 2015). Subsequently, a recent study assessing differences in hair steroid levels due to changes in environmental enrichment also showed differences in hair corticosterone concentrations, confirming that corticosterone as well as other hormones such as dehydroepiandrosterone are easily quantifiable within rat hair (Elmi et al. 2024). It should be noted that as our understanding of hair corticosterone analysis is broadened, the notion that hair corticosterone assessment represents a longer temporal window, serving as a more representative snapshot of long‐term stress, has been challenged (Colding‐Jørgensen et al. 2023). Hair corticosterone concentrations have been found to decrease over time following a stressor, requiring further investigation into the kinetics of hair glucocorticoids. A shave, re‐shave methodology could be employed in future studies, as a means of limiting the observation window and assessing corticosterone over the lifespan of the animal (J. S. Meyer and Novak 2012). Despite new information regarding the time‐frame of study for hair corticosterone, it still represents a longer temporal window than other methods such as faecal corticosterone (~8–12 h) which is more susceptible to immediate stressors such as cage changes that may mask subtle effects (Rowland and Toth 2019). Additionally, some rats do not defecate within a reasonable amount of time, potentially biasing data toward rats with greater anxiety. Thus, analysis of hair corticosterone mitigates such issues and represents a powerful new tool for physiological assessment of rodents.

Assessment of dam behaviour indicated that dams housed in standard cages spent more time in the centre of the open field, indicative of reduced anxiety (Figure 6a); although no differences were found in the elevated plus maze (Figure 6b). Differences observed were also not due to differences in activity, as the distance travelled throughout the duration of the test was insignificant between treatment groups. Interestingly, a similar study found an increase in affective state for dams housed in lofted cages versus standard cages based upon an anticipatory task (Ratuski and Weary 2021). Given the two tests likely assess different aspects of behaviour, it cannot be stated with certainty whether enriched cages provide a significant benefit to dams. An additional consideration for the differences observed may be that the rats utilized within this study were obtained from a breeding facility at GD 14; therefore, their previous housing arrangement was unknown, and they could have undergone additional stress during transport. A potential limitation of this study is the limited assessment of maternal behaviour. Future studies should further assess behaviour of the dams throughout the early stages of pregnancy and the nursing period to determine optimal rearing conditions.

## 5 Conclusion

Within this study, the increased indicators of stress observed in rats reared in enriched cages are expected to be due to the influence of the rat dams' interactions with their pups during development. While previous studies have demonstrated that rats reared in an environment where the mother is manually removed for regular short intervals of time exhibit decreased levels of anxiety during adulthood (Chen and Baram 2016; Lehmann and Feldon 2000), offspring that experience prolonged periods of separation from their mother show indications of inappropriate social behaviour, impaired cognitive development and increased anxiety (Champagne et al. 2003). The ability of the mother to escape her pups as frequently as she chooses results in significant differences in offspring behaviour and physiology; however, whether such changes are beneficial versus detrimental to the offspring requires further investigation. While it is possible that the mother may be spending too much time away from her pups, resulting in more anxious offspring later in life, it is equally likely that the changes observed are not necessarily detrimental to the well‐being of the offspring. Future studies examining the underlying biological or neurobiological mechanisms will aid in further clarifying the relationship between rearing environment and long‐term consequences on both the dam and offspring. A comprehensive understanding of the impacts of rearing environment will be essential for not only optimizing laboratory animal welfare but also improving the reproducibility of animal‐related research.

## Author Contributions

**Logan J. Bigelow:** conceptualization, data curation, formal analysis, investigation, methodology, writing – original draft, writing – review and editing. **Emily K. Pope:** conceptualization, data curation, formal analysis, investigation, methodology, writing – original draft, writing – review and editing. **Carey G. Ousley:** data curation, formal analysis, investigation, writing – review and editing. **Ariana E. McGrattan:** data curation, investigation, writing – review and editing. **Debra S. MacDonald:** investigation, methodology, writing – review and editing. **Paul B. Bernard:** conceptualization, funding acquisition, methodology, project administration, resources, supervision, writing – review and editing.

## Ethics Statement

All procedures performed during this study were conducted in accordance with the guidelines of the Canadian Council on Animal Care and approved by the University of Prince Edward Island Animal Care Committee protocol #21‐032.

## Conflicts of Interest

The authors declare no conflicts of interest.

## Peer Review

The peer review history for this article is available at https://www.webofscience.com/api/gateway/wos/peer‐review/10.1111/ejn.70199.

## Data Availability

Data available upon request from the corresponding author.
